# Beta-Blocker Drug Therapy Reduces Secondary Cancer Formation in Breast Cancer and Improves Cancer Specific Survival

**DOI:** 10.18632/oncotarget.197

**Published:** 2010-10-19

**Authors:** Desmond G. Powe, Melanie J. Voss, Kurt S. Zänker, Hany O. Habashy, Andrew R. Green, Ian O. Ellis, Frank Entschladen

**Affiliations:** ^1^ Department of Cellular Pathology, Queen's Medical Centre, Nottingham University Hospitals NHS Trust, Nottingham, NG7 2UH, UK and The John van Geest Cancer Research Centre School of Science and Technology, Nottingham Trent University, Clifton Lane, Nottingham NG11 8NS UK; ^2^ Institute of Immunology, University of Witten/Herdecke, DE-58448, Witten, Germany; ^3^ School of Molecular Medical Sciences, University of Nottingham, Nottingham, NG7 2UH, UK

**Keywords:** Beta-blockers, cancer, therapy

## Abstract

Laboratory models show that the beta-blocker, propranolol, can inhibit norepinephrine-induced breast cancer cell migration. We hypothesised that breast cancer patients receiving beta-blockers for hypertension would show reduced metastasis and improved clinical outcome. Three patient subgroups were identified from the medical records of 466 consecutive female patients (median age 57, range 28-71) with operable breast cancer and follow-up (>10 years). Two subgroups comprised 43 and 49 hypertensive patients treated with beta-blockers or other antihypertensives respectively, prior to cancer diagnosis. 374 patients formed a non-hypertensive control group. Metastasis development, disease free interval, tumour recurrence and hazards risk were statistically compared between groups. Kaplan-Meier plots were used to model survival and DM. Beta-blocker treated patients showed a significant reduction in metastasis development (p=0.026), tumour recurrence (p=0.001), and longer disease free interval (p=0.01). In addition, there was a 57% reduced risk of metastasis (Hazards ratio=0.430; 95% CI=0.200-0.926, p=0.031), and a 71% reduction in breast cancer mortality after 10 years (Hazards ratio=0.291; 95% CI=0.119-0.715, p=0.007). This proof-of-principle study showed beta-blocker therapy significantly reduces distant metastases, cancer recurrence, and cancer-specific mortality in breast cancer patients suggesting a novel role for beta-blocker therapy. A larger epidemiological study leading to randomised clinical trials is needed for breast and other cancer types including colon, prostate and ovary.

## INTRODUCTION

Although an estimated 38,000 [1] patients are diagnosed with breast cancer in the US each year death rates are declining in part due to adjuvant therapies including the use of ER-antagonists and anti-HER2 (trastuzumab) therapy [2-4]. Nonetheless, approximately 30% of treated BC patients develop distant metastases [5] and these significantly account for 90% of breast cancer deaths [6]. Consequently, therapeutic strategies are needed that target metastasis [7,8].

Metastasis formation involves migration of malignant cells from the primary tumour via lymphatic or blood vessel routes with the process being tightly regulated by exogenous cell signalling molecules, including ligands to G protein-coupled receptors (GPCRs) such as neurotransmitters and chemokines [9,10]. In previous *in vitro* cell migration studies we have shown that the stress catecholamine hormone norepinephrine is a potent inducer of migratory activity in carcinoma cell lines of colon [11], prostate [12], ovarian cancer cells [13] and breast [14] tissue origin, and this finding has been confirmed in a mouse model [15]. Moreover, we have shown that cell migration is mediated by adrenergic receptors (AR) and that the process is inhibited by the beta-blocker adrenergic receptor antagonist drug propranolol which is non-selective for β1AR and β2AR [11,12,15]. Additional support for the therapeutic benefit of beta-blockers is provided by recent studies showing propranolol can reduce proliferation in human pancreatic cell lines [16,17].

Beta-blocker drugs along with ACE-inhibitors, calcium channel antagonists, imidazoline receptor antagonists, and diuretics are clinically well characterized for the therapeutic treatment of hypertension and are proven in reducing life-threatening cerebrovascular events [18,19]. Epidemiologic studies confirm that beta-blocker treatment *per se* has no effect in causing or promoting cancer growth giving reassurance for their continued clinical use [20,21].

In the present study we hypothesised that patients started on and maintained with antihypertensive beta-blocker therapy prior to their breast cancer diagnosis would show reduced distant metastasis formation compared to non-hypertensive breast cancer patients or those treated with other antihypertensive drugs.

## RESULTS

### Clinical Correlations in Patients Treated with Beta-Blocker Drugs compared to other Hypertensive and Non-hypertensive Breast Cancer Patients

Data was obtained for 466 breast cancer patients used in this study and their characteristics are shown in Tables 1a-d. A total of 92 (19.7%) patients had hypertension diagnosed prior to breast cancer diagnosis and were therapeutically treated using a range of antihypertensives including beta-blockers, ACE inhibitors, Ca2^+^ antagonists, imidazoline receptor antagonists or diuretics (Table 2). In particular, 43/92 (46.7%) of this hypertensive subgroup (median age 57 years, range 39-69) received beta-blocker drugs while the remaining 49/92 (53.3%) patients received other antihypertensive drugs. The latter group significantly differed in showing increased median age of 62 years (range 47-70) compared to the non-hypertensive patient subgroup whose median age was 54.5 years (range 28-71). In addition, the non-beta-blocker treated antihypertensive subgroup contained an increased proportion of post-menopausal patients (Table 1c) Otherwise, no significant difference was seen in tumour stage, tumour size, grade, type, vascular invasion, Nottingham Prognostic Index (NPI) or AT between the three patient subgroups.

**Table 1a:** Characteristics for breast cancer patients therapeutically treated with beta-blockers (BB) compared to control breast cancer (BC) patients, excluding patients treated with other types of antihypertensive drugs

Variable	Number (%) Control BC patients	Number (%) BB-treated patients	χ^2^	p-value
**Patients' Age**				
<40	22 (5.9)	1 (2.3)	4.079	0.253
40-50	117 (31.3)	9 (20.9)		
51-60	126 (33.7)	20 (46.5)		
>60	109 (29.1)	13 (30.2)		
**Primary tumoursize**				
≤1.5 cm	134 (35.9)	14 (33.3)	0.111	0.740
>1.5 cm	239 (64.1)	28 (66.7)		
**Tumour stage**				
1	250 (67.0)	31 (73.8)	1.176	0.555
2	89 (23.9)	9 (21.4)		
3	34 (9.1)	2 (4.8)		
**Grade**				
1	75 (20.2)	7 (16.7)	2.205	0.332
2	129 (34.7)	11 (26.2)		
3	168 (45.2)	24 (57.1)		
**NPI**				
Poor	43 (11.6)	6 (14.3)	0.270	0.874
Moderate	212 (57.1)	23 (54.8)		
Good	116 (31.3)	13 (31.0)		
**Development of Recurrence**				
No	220 (59.3)	32 (74.4)	13.091	**0.001**
Positive	151 (40.7)	10 (23.3)		
**Vascular invasion**				
Negative	227 (61.5)	27 (62.8)	1.788	0.409
Probable	40 (10.8)	2 (4.7)		
Definite	102 (27.6)	14 (32.6)		
**Tumour type**				
Ductal/NST	200 (54.6)	27 (62.8)	7.433	0.283
Lobular	42 (11.5)	4 (9.3)		
Tubular and Tubular mixed	78 (21.3)	8 (18.6)		
Medullary	11 (3.0)	1 (2.3)		
Other special types*	10 (2.7)	2 (4.7)		
Mixed**	24 (6.6)	0 (0)		
**Menopausal status**				
Premenopausal	149 (39.8)	9 (20.9)	5.860	**0.015**
Postmenopausal	225 (60.2)	34 (79.1)		

**Table 1b:** Characteristics for breast cancer patients therapeutically treated with beta-blockers (BB) compared to breast cancer (BC) patients treated with other antihypertensive drugs

Variable	Number (%) BC treated with other antihypertensives	Number (%) BB-Treated	χ^2^	p-value
**Patients' Age**				
<40	0 (0)	1 (2.3)	6.507	0.089
40-50	10 (20.4)	9 (20.9)		
51-60	13 (26.5)	20 (46.5)		
>60	26 (53.1)	13 (30.2)		
**Primary tumour size**				
≤1.5 cm	19 (39.6)	14 (33.3)	0.377	0.539
>1.5 cm	29 (60.4)	28 (66.7)		
**Tumour stage**				
1	31 (66.0)	31 (73.8)	0.817	0.665
2	12 (25.5)	9 (21.4)		
3	4 (8.5)	2 (4.8)		
**Grade**				
1	12 (25.0)	7 (16.7)	2.215	0.330
2	16 (33.3)	11 (26.2)		
3	20 (41.7)	24 (57.1)		
**NPI**				
Poor	5 (10.2)	6 (14.3)	1.065	0.587
Moderate	24 (49.0)	23 (54.8)		
Good	20 (40.8)	13 (31.0)		
**Development of Recurrence**				
No	25 (51.0)	32 (74.4)	7.264	**0.026**
Positive	24 (49.0)	10 (23.3)		
**Vascular invasion**				
Negative	30 (61.2)	27 (62.8)	1.811	0.404
Probable	6 (12.2)	2 (4.7)		
Definite	13 (26.5)	14 (32.6)		
**Tumour type**				
Ductal/NST	26 (53.1)	27 (62.8)	8.269	0.309
Lobular	3 (6.1)	4 (9.3)		
Tubular and Tubular mixed	15 (30.6)	8 (18.6)		
Medullary	2 (4.1)	1 (2.3)		
Other special types*	0 (0)	2 (4.7)		
Mixed**	2 (4.1)	0 (0)		
**Menopausal status**				
Premenopausal	12 (24.5)	9 (20.9)	0.165	0.685
Postmenopausal	37 (75.5)	34 (79.1)		

**Table 1c:** Characteristics for breast cancer patients therapeutically treated with antihypertensive drugs (excluding beta blockers) compared to control breast cancer (BC) patients

Variable	Number (%) Control BC patients	Number (%) BC treated with other antihypertensives	χ^2^	p-value
**Patients' Age**				
<40	22 (5.9)	0 (0)	12.708	**0.005**
40-50	115 (30.7)	10 (20.4)		
51-60	127 (34.0)	131 (26.5)		
>60	110 (29.4)	26 (53.1)		
**Primary tumour size**				
≤1.5 cm	134 (35.9)	19 (39.6)	0.246	0.620
>1.5 cm	239 (64.1)	29 (60.4)		
**Tumour stage**				
1	249 (66.9)	31 (66.0)	0.069	0.966
2	89 (23.9)	12 (25.5)		
3	34 (9.1)	4 (8.5)		
**Grade**				
1	74 (19.9)	12 (25.0)	0.709	0.702
2	128 (34.4)	16 (33.3)		
3	170 (45.7)	20 (41.7)		
**NPI**				
Poor	43 (11.6)	5 (10.2)	1.917	0.384
Moderate	213 (57.4)	24 (49.0)		
Good	115 (31.0)	20 (40.8)		
**Development of Recurrence**				
No	218 (58.8)	25 (51.0)	1.063	0.302
Positive	153 (41.2)	24 (49.0)		
**Vascular invasion**				
Negative	228 (61.8)	30 (61.2)	0.057	0.972
Probable	41 (11.1)	6 (12.2)		
Definite	100 (27.1)	13 (26.5)		
**Tumour type**				
Ductal/NST	201 (54.9)	26 (53.1)	12.228	0.093
Lobular	42 (11.5)	3 (6.1)		
Tubular and Tubular mixed	78 (21.3)	15 (30.6)		
Medullary	11 (3.0)	2 (4.1)		
Other special types*	9 (2.5)	0 (0)		
Mixed**	24 (6.6)	2 (4.1)		
**Menopausal status**				
Premenopausal	147 (39.3)	12 (24.5)	4.053	**0.044**
Postmenopausal	229 (60.7)	37 (75.5)		

**Table 1d:** Characteristics for breast cancer patients therapeutically treated with beta-blockers (BB) compared to all other patients including those receiving other antihypertensive drug treatment

Variable	Number (%) All other patients	Number (%) BB-Treated	χ^2^	p-value
**Patients' Age**				
<40	22 (5.2)	1 (2.3)	3.926	0.270
40-50	127 (30.1)	9 (20.9)		
51-60	139 (32.9)	20 (46.5)		
>60	135 (31.9)	13 (30.2)		
**Primary tumour size**				
≤1.5 cm	153 (36.3)	14 (33.3)	0.150	0.699
>1.5 cm	268 (63.7)	28 (66.7)		
**Tumour stage**				
1	281 (66.9)	31 (73.8)	1.190	0.552
2	101 (24.0)	9 (21.4)		
3	38 (9.0)	2 (4.8)		
**Grade**				
1	87 (20.7)	7 (16.7)	2.368	0.306
2	145 (34.5)	11 (26.2)		
3	188 (44.8)	24 (57.1)		
**NPI**				
Poor	48 (11.4)	6 (14.3)	0.305	0.859
Moderate	236 (56.2)	23 (54.8)		
Good	136 (32.4)	13 (31.0)		
**Development of Recurrence**				
No	244 (58.2)	33 (78.6)	6.854	**0.010**
Positive	175 (41.8)	9 (21.4)		
**Vascular invasion**				
Negative	257 (61.5)	27 (62.8)	1.877	0.391
Probable	46 (11.0)	2 (4.7)		
Definite	115 (27.5)	14 (32.6)		
**Tumour type**				
Ductal/NST	233 (55.5)	28 (65.1)	3.534	0.832
Lobular	46 (10.9)	4 (9.3)		
Tubular and Tubular mixed	92 (21.9)	7 (16.3)		
Medullary	13 (3.1)	1 (2.3)		
Other special types*	10 (2.4)	2 (4.6)		
Mixed**	26 (6.2)	1 (2.3)		
**Menopausal status**				
Premenopausal	160 (37.9)	11 (25.6)	2.553	0.110
Postmenopausal	262 (62.1)	32 (74.4)		
*Includes Mucoid, invasive cribriform and invasive papillary carcinoma. ** Includesductal/NST mixed with lobular or special types.				

**Table 2:** Beta-blocker and other therapeutic drugs were used to treat breast cancer patients for pre-existing hypertension

Drug	Patient Numbers
**Beta-blockers**	
Atenolol	25
Propranolol	7
Bisoprolol	7
Timolol	4
Subtotal	43
**Other drug treatments**	
Captopril (ACE-Inhibitor)	6
Ramipril (ACE-Inhibitor)	4
Enalapril (ACE-Inhibitor)	3
Lisinopril (ACE-Inhibitor)	3
	
Nifedipine (Ca2+ antagonist)	5
Amlodipine (Ca2+ antagonist)	5
Nicardipine (Ca2+ antagonist)	1
	
Moxonidine (Imidazoline receptor antagonist)	1
	
Bendrofluazide (Diuretic)	21
Subtotal	49
**Total**	**92**

Patients receiving beta-blockers showed a significant reduction in formation of distant metastasis (χ^2^=4.986, p=0.026) and tumour recurrence (χ^2^=13.091, p=0.001) compared to non-hypertensive BC control patients (Table 3).

**Table 3:** Two hypertensive breast cancer (BC) patient groups comprising those treated with beta-blockers and non-beta blocker antihypertensive drugs were compared with a non-hypertensive non-treated BC group for formation of distant metastases

Metastasis formation	Treatment in breast cancer groups		χ^2^	P-value
	Non-hypertensive patients (%)	Beta-blocker treated hypertensives (%)		
Negative	271 (72.7)	38 (88.4)	4.986	0.026
Positive	102 (27.3)	5 (11.6)		

	**Other treated anti-hypertensive patients (%)**	**Beta-blocker treated patients (%)**		
Negative	34 (69.4)	38 (88.4)	4.852	0.028
Positive	15 (30.6)	5 (11.6)		

	**Non hypertensive BC patients (%)**	**Other anti-hypertensive patients**		
Negative	271 (72.7)	34 (69.4)	0.231	0.631
Positive	102 (27.3)	15 (30.6)		

### Hypertensive Breast Cancer Patients Treated with Beta-Blockers Compared with Other Antihypertensive Drugs

Beta-blocker treated breast cancer patients differed significantly to patients receiving other antihypertensives (χ^2^=4.852, p=0.028) in showing reduced development of distant metastasis (5/43 (11.6%)) compared to their counterparts (15/49 (30.6%)) (Table 3). In addition, the beta-blocker treated subgroup showed significantly reduced tumour recurrence (χ^2^=7.264, p=0.026; Table 1b).

## PATIENT OUTCOME

### Univariate analysis:

**Figure 1:**
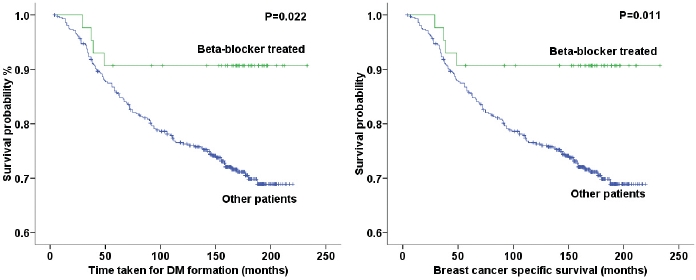
a: Hypertensive BC patients therapeutically treated with beta-blockers showed significantly (p=0.022) longer times before acquiring metastases compared to non-treated patients b. Hypertensive BC patients receiving beta-blocker therapy showed significantly (p=0.011) improved 10 year survival rates compared to non-treated patients.

Kaplan-Meier modelling with log rank testing showed beta-blocker treated patients had longer distant metastasis-free interval (Log rank (LR)=5.208, p=0.022) (Fig 1a) and longer breast cancer specific survival (LR=6.479, p=0.011) (Fig 1b), and longer disease free interval (LR=6.658, p=0.011) when compared to non-treated breast cancer patients.

### Multivariate analysis:

A multivariate Cox hazard model was used to determine the hazard ratio (HR) for predicting breast cancer specific survival and distant metastasis risk in patients receiving beta-blocker treatment compared to other significant breast cancer variables including tumour size, stage and grade. Patients receiving beta-blocker treatment had a 71% reduced risk of cancer-associated mortality (HR=0.291, CI= 0.119-0.715, p=0.007). In addition, beta-blocker treated patients showed a 57% risk reduction in developing distant metastasis (HR=0.430, CI= 0.200-0.926, p=0.031; Table 4) compared to non-treated breast cancer patients.

**Table 4:** The effect of beta-blocker treatment on breast cancer specific survival (BCSS) and distant metastasis (DM) formation was compared with tumour size, grade and stage to determine the relative risk (Hazard Ratios (HR)) in BC patients

Parameter	HR	p-value	95% Confidence Interval	
			Lower	Upper
**BCSS**				
Tumour size Tumour grade Tumour stage beta-blocker treatment	1.985	0.004	1.248	3.159
	1.904	<0.001	1.435	2.526
	1.565	<0.001	1.218	2.011
	0.291	0.007	0.119	0.715
**DM**				
Tumour size Tumour grade Tumour stage beta-blocker treatment	1.916	0.005	1.221	3.005
	1.519	0.002	1.171	1.971
	1.624	<0.001	1.270	2.076
	0.430	0.031	0.200	0.926

## DISCUSSION

### Evidence of the biological mechanism between stress hormones and tumour cell migration

Tumour metastasis is a complex process and is associated with generally poor clinical outcome. Therapeutic strategies are needed that can prevent its occurrence, thereby prolonging patient life. The present study was performed to validate the putative role of beta-blocker adrenergic receptor antagonists in retarding the progress of breast cancer disease by reducing metastasis formation. We performed an epidemiological study of breast cancer patients with long term clinical follow up (>10 years) and showed that patients receiving antihypertensive beta-blocker drugs significantly benefit by a 57% reduction in distant metastasis formation and a 71% reduced risk of dying from breast cancer compared to control patients.

It is increasingly being recognised that stress can promote cancer progression through an indirect effect on the immune system [22]. Moreover, a biological mechanism of action has been proposed for the involvement of catecholamine stress hormones. It has been shown that norepinephrine can directly stimulate tumour cell migration and this effect is mediated by the beta-adrenergic receptor, β2AR. High levels of β_2_AR have been reported in human cell lines [11,23,24] and tumour samples [25] and importantly, we have shown that cell migration in a number of cancer models is inhibited by the beta-blocker adrenergic receptor antagonist, propranolol [11,12,14].

### Proof-of principle epidemiological pilot study shows beta-blocker drugs reduce metastasis and tumour recurrence in patients with breast cancer, leading to an improvement in survival

The aim of the current study was to translate these findings into a clinical setting by testing the hypothesis that breast cancer patients receiving beta-blockers for pre-existing hypertension would show a significant reduction in tumour metastasis with a consequent reduction in mortality.

In line with expectations, antihypertensive drug treatment was more frequently prescribed in older post-menopausal patients with no significant difference seen between the beta-blocker and other antihypertensive treatment subgroups. The beta-blocker treated subgroup was shown to have significantly (57%) reduced risk of developing distant metastasis and tumour recurrence compared to patients receiving either no, or other types of, antihypertensive drugs. As a consequence of this, beta-blocker treated patients showed a significant increase in breast cancer specific survival and increased disease free interval. To the best of our knowledge, this is the first report highlighting the *in vivo* therapeutic benefits of beta-blockers in breast cancer patients, supporting the biological mechanism of norepinephrine-induced cell migration mediated by adrenergic receptor activation as shown in cell lines and animal models. These findings support recent epidemiological cancer studies that have shown adrenoceptor antagonists have the potential for providing a novel clinically effective therapeutic strategy in treating several different cancer types. Beta-blockers have been shown to reduce the incidence of endocrine-regulated prostate cancer [26], but this beneficial effect is not just limited to inhibition of beta receptors because patients receiving alpha antagonists also show a reduced incidence of prostate [27] and bladder [28] cancer. Moreover, a generalised reduction in all cancer types has been reported in beta-blocker treated patients [29].

### Study limitations

Several limitations apply to our epidemiological study including patient population size and factors that were not controlled for in using an existing patient dataset. In mitigation, the beta-blocker and other hypertensive-treated cohort were of approximately the same size and together accounted for approximately 20% of the total patient cohort. In addition, all patient subgroups were evenly matched for adjuvant therapy and age making it unlikely that this was the reason for the significant benefits seen in the beta-blocker group. A further possible limitation is that it is unknown (and impossible to establish) how long patients had breast cancer prior to a formal diagnosis, and so the duration required for beta-blockers to prevent development of metastases can not be calculated from this type of study. Some of these limitations can be addressed by performing a much larger epidemiological study, leading onto a randomised controlled clinical trial. The latter would determine a) If beta-blocker drugs can be used as a prophylactic for metastatic formation in breast cancer, b) The optimal adjuvant therapy dosage in breast cancer, c) Whether beta-blocker treatment is effective in clinically treating patients with other types of cancer, e.g. prostate, pancreatic and colonic cancer, suggested by our cell line models.

### Future studies

The current study suggests that adrenoceptor antagonists have the potential for retarding breast cancer progression and improving clinical outcome. Additional studies are needed to assess the protein expression of adrenoceptors in breast and other cancer types to test if they can be used as prognostic and predictive biomarkers in determining clinical outcome and likely response to antagonist treatment.

## MATERIALS AND METHODS

### Patient selection

Therapeutic drug and medical history was obtained for 466 patients with stage I and II primary operable breast carcinoma, aged 71 years or less, who presented consecutively to the Nottingham City Hospital between 1987 and 1994 as previously reported [30]. Patients were placed into one of three subgroups according to whether they received (1) beta-blocker treatment for hypertension, (2) other antihypertensive drug treatment, or were (3) normotensive. To qualify for subgroup (1) or (2) membership, patients needed to have been receiving antihypertensive therapy for at least 1 year prior to breast cancer diagnosis. This criterion was applied to minimise differences due to length of drug treatment; patients that received hypertensive drugs for less than 1 year were excluded because the primary objective tested was that beta-blockers may have a role in preventing metastasis formation in early stage breast cancer rather than eradicating or neutralising established primary and secondary cancers.

Patient's clinical and pathologic data was available including age, histologic tumour type, primary tumour size, lymph node status and histologic grade, Nottingham prognostic index (NPI), vascular invasion (VI), and radio/chemotherapy. Patients were considered for adjuvant therapy (AT) in a standardised scheduled on the basis of prognostic and predictive factor status including Nottingham Prognostic Index (NPI) [31], oestrogen receptor-α (ERα) status, and menopausal status. Patients with a good prognostic index (NPI ≤ 3.4) did not receive AT. Hormonal therapy (HT) was prescribed to patients with ERα+ tumours and NPI scores of >3.4 (moderate and poor prognostic groups). Pre-menopausal patients within the moderate and poor prognostic groups were candidates for CMF (Cyclophosphamide, Methotrexate, and 5-Flourouracil) chemotherapy; patients with ERα+ tumour were also offered HT. Conversely, postmenopausal patients with moderate or poor NPI and ERα+ were offered HT, while ERα-patients received CMF. Data has been accrued on a prospective basis for breast cancer specific survival (BCSS), disease free interval (DFI), formation of distant metastases (DM) and local tumour recurrence. BCSS was defined as the time (in months) from the date of the primary surgical treatment to the time of death from breast cancer. DFI was defined as the interval (in months) from the date of the primary surgical treatment to the first locoregional or distant metastasis. Mean follow-up time was 124 months for the study cohort.

### Univariate and Multivariate Statistics

The clinical outcome in three patient cohorts (beta-blocker drug treated, other antihypertensive drug treated, and non-hypertensive breast cancer groups) was tested using Kaplan-Meier plots with log rank test to assess significance including breast cancer specific survival, disease free interval, and distant metastasis formation. Other associations including tumour recurrence was tested using Chi square or Fishers exact test. Multivariate Cox regression analysis was used to evaluate the hazard ratio and any independent prognostic effect of the variables using 95% confidence interval (Version 15, SPSS Inc, IL, USA). A p-value of <0.05 was considered significant.
